# Prevalence of antibiotic resistant *Staphylococcus aureus* from raw milk samples collected from the local vendors in the region of Tirupathi, India

**DOI:** 10.14202/vetworld.2015.478-481

**Published:** 2015-04-12

**Authors:** Sudhanthirakodi Sudhanthiramani, Chinta Siva Swetha, Sukumar Bharathy

**Affiliations:** Department of Veterinary Public Health and Epidemiology, College of Veterinary Science, Sri Venkateshwara Veterinary University Campus, Tirupati, Andhra Pradesh - 517 502, India

**Keywords:** antibiotic resistant, local milk vendor, milk, *Staphylococcus aureus*

## Abstract

**Aim:**

The study was carried out with the aim to identify the suitability of the milk for consumer use with special reference to *Staphylococcus aureus* from milk samples collected from various local vendors and determine the antibiotic susceptibility pattern of those positive isolates.

**Materials and Methods:**

A total of 110 milk samples were collected from the local milk vendors in and around Tirupathi region of India. All the samples were enriched with buffered peptone water in 9:1 ratio and the then inoculated on baird parker agar medium with added 2% egg yolk tellurite emulsion as selective medium for *S. aureus* and confirmed with mannitol salt agar, Gram’s staining and biochemical tests. The typical cultural characters with coagulase-positive samples were taken as positive samples the positive samples were tested for antibiotic susceptibility with 10 different antibiotics by employing disc diffusion method.

**Results:**

Prevalence of coagulase-positive *S. aureus* was 39.09% (43/110) from the milk samples. The antibiotic susceptibility test of positive isolates showed high resistant toward penicillin G 37/43 (86.04%) and ampicillin 32/43 (74.42%), and also showed resistant to methicillin 6/43 (13.95%), cephalothin 6/43 (13.95%), tetracycline 6/43 (13.95%), ciprofloxacin 4/43 (9.30%), enrofloxacin 3/43 (6.97%), cefoxitin 2/43 (4.65%), gentamicin 2/43 (4.65%), and co-trimoxazole 2/43 (4.65%). Many individual isolates showed resistant against two or more antibiotics in our study.

**Conclusion:**

The above study results show that the milk samples collected from local vendor having *S. aureus*, which can induce disease condition as well as antibiotic resistant to the humans particularly young children and old age peoples by means of consumption of raw milk and its products. This is the public health issue, which needs to be solved by educating the local vendors regarding health problems related to unhygienic milk supply and make the awareness among the consumers about this hazards and preventive measures.

## Introduction

There are so many people still purchasing the milk from the local milk vendors for day to day consumption, which is unpasteurized. Many people in the Tirupathi municipality region of India believe that this unpasteurized milk from local vendors having more nutritive value than pasteurized milk. Due to increased cost of pasteurized milk, milk from the local vendors itself considering as a main source of milk for daily consumption. This factor may lead to so many disease outbreaks which can transmit through milk, if the milk was not boiled completely or consumed raw. *S. aureus* is most common causative organism for subclinical mastitis in cows and the cow udder itself give optimum temperature for growth of this organism [1].

When this subclinical mastitis milk mixed with normal milk may cause *S. aureus* food poisoning. Milk is the main source of nutrient for growth of most of the microorganisms as it having all the essential nutrients [2,3]. *S. aureus* causes the foodborne intoxication symptoms in patients such as sudden onset of nausea, vomiting, abdominal cramps, and diarrhea [4], including severe skin and other diseases, pneumonia, and septicemia [5]. Milk can act as a vehicle for transmitting the *S. aureus* from animal to cause the severe foodborne intoxication in human. It has been recorded that *S. aureus* causes the subclinical mastitis and contaminate the udder and milk; acting as the main source of contaminants. Contaminated milking equipment’s and the milker’s hands also may be the source of infection [6-8].

Each strain of *S. aureus* has ability to produce so many virulence factors, which includes foodborne intoxication producing enterotoxins (SEA to SEE and SEG to SEQ) and exfoliative toxin A and B, and toxic shock syndrome toxin. Pasteurization may kill the *S. aureus*, but the thermostable *Staphylococcal* enterotoxinss will be holding the biological activity and produce food intoxication in human [9,10]. *Staphylococcus* foodborne intoxication cases reported in a variety of values from different places, food intoxication by using raw milk itself account for many cases of *Staphylococcus* foodborne intoxication [11]. Apart from the foodborne intoxication, antibiotic resistance of *S. aureus* against the common antibiotics is the greatest public health issues everywhere. *S. aureus* shows very high resistance against Penicillin, vancomycin, and methicillin compared to other antibiotics [12,13]. Milk from the local vendors with a high percentage of *S. aureus* and its antibiotic resistance may lead to very serious public health issues.

The study was carried out with the aim to identify the suitability of the milk for the consumers use with special reference to *S. aureus* from milk samples collected from various local vendors and determine the antibiotic susceptibility pattern of those positive isolates.

## Materials and Methods

### Ethical approval

There were no live animals used in this study, so there is no ethical approval necessary.

### Sampling

A total of 110 milk samples were collected from different local vendors, who are selling the milk in and around the region of Tirupathi, India. This milk samples were collected in a sterile containers (Hi Media) and transported to the laboratory under chilling condition by the use of ice bags. Approximately, 250 ml of milk samples were collected from each vendor. All the samples were processed on the same day of sample collection itself.

### Isolation and identification of *S. aureus* from milk samples

All the milk samples were pre-enriched with buffer peptone water at 9:1 ratio (9 parts of buffered peptone water and 1 part of milk sample) and incubated at 37°C for 18-24 h as per standard protocol. After incubation a loop full of inoculum was streaked onto Baird Parker agar (Hi Media) supplemented with egg yolk and tellurite emulsion (2%) (Hi Media) and incubated at 37°C for 24 h. A black shiny colony with halo zone was chosen as a positive *S. aureus* colony and further processed for the confirmation [14,15]. The typical colonies from Baird Parker agar were transferred to mannitol salt agar (MSA) and nutrient agar (NA), incubated at 37°C for 24 h. From the NA plates, the colonies were further used for other biochemical tests like catalase test, oxidase test, and coagulase test with sheep blood plasma. *S. aureus* from other species of *Staphylococcus* was differentiated by colony characters on MSA. The *S. aureus* colonies on MSA were golden yellow in color. All the biochemical test and MSA colony characters were combined and confirmed as *S. aureus* [16,17].

Biochemically confirmed *S. aureus* isolates were further tested for antibiotic susceptibility patterns by using common antibiotic disc like, penicillin G, ampicillin, methicillin, tetracycline, enrofloxacin, co-trimoxazole, streptomycin, cephalothin, cefixime, and ciprofloxacin by disc diffusion method [18].

## Results

The present study revealed that the prevalence of *S. aureus* was 39.09% (43/110) in the milk samples collected from local vendors in and around the region of Tirupathi.

**Figure-1:**
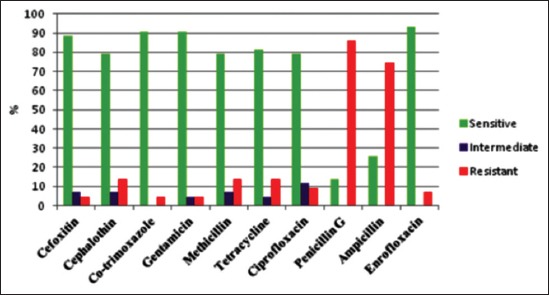
Antibiotic sensitivity pattern for *Staphylococcus aureus*.

Antibiotic resistant patterns of the positive isolates of *S. aureus* in the current study showed various result with highest percentage of antibiotic resistant to penicillin G (86.04%) followed by ampicillin (74.42%), and 13.95% of positive isolates were resistant to methicillin, tetracycline, and cephalathin, 9.3% resistant to ciprofloxacin, 6.97% resistant to enrofloxacin, and 4.65% resistant to cefoxitin, gentamicin, and co-trimaxazole (Table-1 and Figure-1).

**Table-1:** Antibiotic susceptibility patterns of positive isolates of *S. aureus* from milk samples.

Antibiotics	Susceptible (%)	Intermediate (%)	Resistant
Cefoxitin	38 (88.37)	3 (6.97)	2 (4.65)
Cephalothin	34 (79.07)	3 (6.97)	6 (13.95)
Co-trimoxazole	41 (90.34)	0 (0)	2 (4.65)
Gentamicin	39 (90.69)	2 (4.65)	2 (4.65)
Methicillin	34 (79.07)	3 (6.97)	6 (13.95)
Tetracycline	35 (81.39)	2 (4.65)	6 (13.95)
Ciprofloxacin	34 (79.07)	5 (11.63)	4 (9.3)
Penicillin G	6 (13.95)	0 (0)	37 (86.04)
Ampicillin	11 (25.58)	0 (0)	32 (74.42)
Enrofloxacin	40 (93.02)	0 (0)	3 (6.97)

*S. aureus=Staphylococcus aureus*

Of 43 positive isolates of *S. aureus* 32 (74.42%) isolates showed resistant towards two or more antibiotics (Table-2).

**Table-2:** Anti-drug profile of isolates.

Antibiotic resistant profile	No. of isolates (*n*=43)	Percentage of isolates (*n*=43) (%)
No resistant	6	13.95
One	5	11.63
Two	16	37.21
Three	6	13.95
Four	8	18.60
Five	1	2.33
Eight	1	2.33

## Discussion

Milk is the nutritious food for newborn animals as well as human. It’s having essential nutrition for the growth and maintenance of good health. At the same time, milk is more prone to harbor the microorganisms as it having all the essential components which are useful for the growth and multiplication of the microorganisms [19,20].

The results of present prevalence study in and around Tirupathi region of India and other author’s results from various parts of the world are differing vastly. Even from different regions of India also the prevalence rates were differing. However, results of study results at Tirupathi region showed prevalence of 39.09%, which were very high compared to other studies conducted by Thaker *et al*. [9], who reported 6.25% in Gujarat region of North India and Kumar *et al*. [15] reported 26% prevalence in milk samples collected from local vendors of Pantnagar, India. Sarkar, *et al*. [21] documented 74.5% (149/200) of the milk samples were positive for *S. aureus* from the Karnal, North India, and Lingathurai *et al*. [22] reported 61.7% of prevalence of *S. aureus* from 60 raw milk samples from Madurai region of South India; it is higher than our study and lesser than Thaker *et al*. [19] study report.

Results obtained from this study were more or less when compared with the prevalence rates that reported by various authors in different tropical and temperate countries. Prevalence rate from morocco and Palestine reported by Bendahon *et al*. [23] and Farhan *et al*. [24] as 40% and 36.9%, respectively, which were closer to this study. However, lower prevalence has been previously reported by Fagundes *et al*. [25] (10.8%) from São Paulo state, Brazil, Ayano *et al*. [26] (13.8%) from Holeta, Ethiopia, Ekici *et al*. [27] (18.18%) from Turkey, and D’Amico *et al*. [28] (29%) from Vermont, United States. From all these study results of above mentioned indicates prevalence of *S. aureus* is varied from place to place and regions to regions around the world and it highlights that hygienic practice of milking and selling influence the prevalence of *S. aureus* in milk.

Holm *et al*. [29] and Leonard *et al*. [30], reported in general, milk in the udder usually a sterile one, but during unhygienic maintenance of animals shed, milking practice, disease conditions may lead to entry of the microorganisms in to the udder. Once the microbe’s enters inside the udder will cause the different disease conditions, in which mastitis is one of the most common. Most cases of clinical and sub-clinical mastitis caused by *S. aureus*, and it will be transmitted through milk. Contamination with *S. aureus* may also come through improper sanitary management of farm animals, dirty udder of the milking animals, milking vessels, and the milk handlers.

As per the statement mentioned the above by authors, we do believe that the local vendors of Tirupathi region may mix the mastitis milk and normal milk together with or without their knowledge. Even the inadequate cleaning, improper sanitation in the farm or milking equipment’s, sick milking man or sellers are the other factors may be the responsible for this prevalence of *S. aureus* in milk samples.

According to the obtained results of antibiotic sensitivity tests on the *S. aureus* isolates from raw milk was varied from previous study conducted by Thaker *et al*. [19], In this study, the isolates were sensitive to co-trimoxazole 95.34%, methicillin, and cephalothin 79.07%, ampicillin 25.58%, whereas in Thaker *et al*. study; it was 100%, 100%, 100% and 60%, respectively. This difference in the results of various study indicates antibiotic resistant pattern of *S. aureus* changing. By means of different mechanisms, *S. aureus* developing resistant to different antibiotics day by day. Isolation of antibiotic resistant *S. aureus* from milk samples against these drugs poses a major challenge in human medicine because these drugs are commonly used in the treatment of human.

From the current study, it is noted that co-trimoxazole, gentamicin, cefoxitin, enrofloxacin were moderately effective against *S. aureus*. Still no drug achieved 100% susceptibility in this study, if the necessary action not taken against indiscriminate use of antibiotics, prevalence of antibiotic resistant *S. aureus* may increase further, it may lead to serious health hazards to humans.

## Conclusion

In this preliminary study, *S. aureus* were isolated from 39.09% (43/110) of raw milk samples collected from local vendors, this is clearly indicates that there is a possibility of potential public health threat through consumption of milk and milk product. All these isolates presented multiple drug resistance for more than two drugs. The higher percentage of multi-drug resistance pattern in this study indicates alarming situation for designing prevention and control measures. The presence of *S. aureus* in milk samples that collected from various local vendors indicates milk may be contaminated with mastitis milk or may be due to unhygienic practice and these milk samples only they are selling to this Tirupathi region directly without any processing. This kind of practice will may leads to serious public health issues like food borne intoxication, or transfer of antibiotic resistant *S. aureus* to the human population. This issue has to be taken as an important public health problem by the local authorities and there is a need of impose continuous surveillance of milk quality for providing better health to the consumers.

## Authors’ Contributions

SS and SB supervised research work. SS, SB, and CSS carried out sample collection, bacterial isolation, and ABST. All authors contributed drafting and revision of the manuscript. All authors read and approved the final manuscript.

## References

[1] Adesiyun A.A, Webb L.A, Romain H.T (1998). Prevalence and characteristics of *Staphylococcus aureus* strains isolated from bulk and composite milk and cattle handlers. J. Food Prot.

[2] Soomro A.H, Arain M.A, Khaskheli M, Bhutto B (2003). Isolation of *Staphylococcus aureus* from milk products sold at sweet meat shops of Hyderabad. Online J. Biol. Sci.

[3] Zakary E.M, Nassif M.Z, Mohammed G.M (2011). Detection of *Staphylococcus aureus* in bovine milk and its product by real time PCR assay. Glob. J. Biotech. Biochem.

[4] Dzirba W.K, Osek J (2011). Identification of genes encoding classical staphylococcal enterotoxins in *Staphylococcus aureus* isolated from raw milk. Bull. Vet. Inst. Pulway.

[5] Lowy F.D (1998). *Staphylococcus aureus* infection. N. Engl. J. Med.

[6] Capurro A, Concha C, Nilsson L, Ostensson K (1999). Identification of coagulase positive *Staphylococci* isolated from bovine milk. Acta Vet. Scand.

[7] Bonfoh B, Wasem A, Traore A.N, Fane A, Spillmann H, Simbe C.F, Alfaroukh I.O, Nicolet J, Farah Z, Zinsstag J (2003). Microbiological quality of cow’s milk taken at different intervals from the udder to the selling point in Bamako (Mali). Food Control.

[8] De Oliveira L.P, Soares e, Barros L.S, Silva V.C, Cirqueira M.G (2011). Study of *Staphylococcus aureus* in raw and pasteurized milk consumed in the Reconcavo area of the State of Bahia, Brazil. J. Food Protect. Technol.

[9] Thaker H.C, Brahmbhatt M.N, Nayak J.B (2013). Isolation and identification of *Staphylococcus aureus* from milk and milk products and their drug resistance patterns in Anand, Gujarat. Vet. World.

[10] Bergdoll M.S, Easman C.S.F, Adlam C (1983). Enterotoxins. *Staphylococci* and *Staphylococcal* Infections.

[11] Jayarao B.M, Pillai S.R, Sawant A.A, Wolfgang D.R, Hegde N.V (2004). Guidelines for monitoring bulk tank milk somatic cell and bacterial counts. J. Dairy Sci.

[12] Fagundes H, Oliveira C.A.F (2004). Infecções intramamárias causadas por *Staphylococcus aureus* e suas implicações em Saúde Pública. Cienc. Rural.

[13] Kitara L.D, Anywar A.D, Acullu D, Odongo-Aginya E, Aloyo J, Fendu M (2011). Antibiotic susceptibility of *Staphylococcus aureus* in suppurative lesions in Lacor Hospital, Uganda. Afr. Health Sci.

[14] Karmi M (2013). Prevalence of methicillin-resistant *Staphylococcus aureus* in poultry meat in Qena, Egypt. Vet. World.

[15] Kumar R, Prasad A (2010). Detection of *E. coli* and *Staphylococcus* in milk and milk products in and around Pantnagar. Pak. J. Nutr.

[16] Singh P, Prakash A (2008). Isolation of *Escherichia coli*, *Staphylococcus aureus* and *Listeria monocytogenes* from milk products sold under market conditions at Agra Region. Acta Agric. Slov.

[17] Addis M, Pal M, Kyule N (2011). Isolation and identification of staphylococcus species from raw bovine milk in Debre Zeit, Ethiopia. Vet. Res.

[18] Bauer A.W, Kirby W.M.M, Sherris J.C, Turck M (1966). Antibiotic susceptibility testing by a standardized single disk method. Am. J. Clin. Pathol.

[19] Oliver S.P, Jayarao B.M, Almeida R.A (2005). Foodborne Pathogens, Mastitis, Milk Quality and Dairy Food Safety, NMC Annual Meeting Proceedings.

[20] Asperger H, Zanger P, Roginski H, Fuquay J.W, Fox P.F (2003). Staphylococcus aureus. Encyclopedia of Dairy Sciences.

[21] Sarkar P, Mohanta D, Debnath C (2014). *Staphylococcus aureus* in dairy animals and farm workers in a closed herd in Karnal, North India: Assessment of prevalence rate and COA variations. Int. J. Innov. Res. Sci. Eng. Technol.

[22] Lingathurai S, Vellathurai P (2011). Bacteriological quality and safety of raw cow milk in Madurai, South India. Webmed. Cent. Microbiol.

[23] Bendahon A, Lebbadi M, Ennanei L, Essadqui F.Z, Abdin M (2008). Characterization of *Staphylococcus species* isolation from raw milk and milk products (iIben and jben) in North Marocco. J. Infect. Dev. Ctries.

[24] Farhan M, Salk S (2007). Evaluation of bacteriological contamination in raw (unprocessed) milk sold in different regions of Lahore (Pakistan). J. Agric. Soc. Sci.

[25] Fagundes H, Barchesi L, Filho AN, Ferreira L.M, Oliveira C.A.F (2010). Occurrence of *Staphylococcus aureus* in raw milk produced in dairy farms in São Paulo state, Brazil. Braz. J. Microbiol.

[26] Ayano A.A, Hiriko F, Simyalew A.M, Yohannes A (2013). Prevalence of subclinical mastitis in lactating cows in selected commercial dairy farms of Holeta district. J. Vet. Med. Anim. Health.

[27] Ekici K, Bozkurt H, Isleyici O (2004). Isolation of some pathogens from raw milk of different milch animal. Pak. J. Nutr.

[28] D’Amico D.J, Donnelly C.W (2010). Microbiological quality of raw milk used for small-scale artisan cheese production in vermont: Effect of farm characteristics and practices. J. Dairy Sci.

[29] Holm C, Jespersen L (2003). A flow-cytometric gram-stainung technique for milk associated bacteria. Appl. Environ. Microbiol.

[30] Leonard F.C, Markey B.K (2008). Meticillin-resistant *Staphylococcus aureus* in animals: A review. Vet. J.

